# Neurological involvement among non-hospitalized adolescents and young adults 6 months after acute COVID-19

**DOI:** 10.3389/fneur.2024.1345787

**Published:** 2024-02-07

**Authors:** Lise Beier Havdal, Joel Selvakumar, Lise Lund Berven, Tonje Stiansen-Sonerud, Henrik Zetterberg, Kaj Blennow, Trygve Holmøy, Vegard Bruun Bratholm Wyller

**Affiliations:** ^1^Department of Paediatrics and Adolescent Health, Akershus University Hospital, Lørenskog, Norway; ^2^Institute of Clinical Medicine, University of Oslo, Oslo, Norway; ^3^Department of Clinical Molecular Biology (EpiGen), University of Oslo and Akershus University Hospital, Lørenskog, Norway; ^4^Institute of Neuroscience and Physiology, Department of Psychiatry and Neurochemistry, Sahlgrenska Academy, University of Gothenburg, Mölndal, Sweden; ^5^Clinical Neurochemistry Laboratory, Sahlgrenska University Hospital, Mölndal, Sweden; ^6^UCL Institute of Neurology, Department of Neurodegenerative Disease, Queen Square, London, United Kingdom; ^7^UK Dementia Research Institute, London, United Kingdom; ^8^Hong Kong Center for Neurodegenerative Diseases, Kowloon, Hong Kong SAR, China; ^9^Department of Neurology, Akershus University Hospital, Lørenskog, Norway

**Keywords:** COVID-19, neurofilament, glial fibrillary acidic protein, post-COVID-19 condition, adolescents, cognitive functions, fatigue

## Abstract

**Introduction:**

The post-COVID-19 condition (PCC) is characterized by debilitating persistent symptoms, including symptoms suggesting neurological aberrations such as concentration difficulties, impaired memory, pain, and sleep disturbances. The underlying mechanisms remain elusive. This study aimed to investigate brain injury biomarkers, neurocognitive test performance, and self-reported neurological and neuropsychological symptoms in young people with PCC.

**Methods:**

A total of 404 non-hospitalized adolescents and young adults aged 12–25 years who tested positive for SARS-CoV-2, along with 105 matched SARS-CoV-2 negative individuals, were prospectively enrolled and followed-up for 6 months (Clinical Trials ID: NCT04686734). All participants underwent comprehensive assessment encompassing clinical examinations, questionnaires, neurocognitive testing and blood sampling. Serum samples were immunoassayed for the brain injury biomarkers neurofilament light chain (Nfl) and glial fibrillary acidic protein (GFAp). At 6 months, cross-sectional analyses of serum Nfl/GFAp, neurocognitive test results and symptom scores were performed across groups based on adherence to PCC criteria as well as initial SARS-CoV-2 test results. Also, associations between Nfl/GFAp, neurocognitive test results, and symptom scores were explored.

**Results:**

A total of 381 SARS-CoV-2 positive and 85 SARS-CoV-2 negative were included in the final analysis at 6 months, of whom 48% and 47%, respectively, adhered to the PCC criteria. Serum levels of Nfl and GFAp were almost equal across groups and did not differ from reference values in healthy populations. Also, neurocognitive test results were not different across groups, whereas symptom scores were significantly higher in patients fulfilling PCC criteria (independent of initial SARS-CoV-2 status). No significant associations between Nfl/GFAp, neurocognitive test results, and symptom scores were found.

**Conclusion:**

Normal brain injury biomarkers and neurocognitive performance 6 months after mild COVID-19 implies that the persistent symptoms associated with PCC are not concurrent with ongoing central nervous system damage or permanent disruption of cognitive functions. This finding contradicts the notion of neuroinflammation as a likely explanation for the persistent symptoms.

## Introduction

The majority of individuals infected with Severe Acute Respiratory Syndrome Coronavirus 2 (SARS-CoV-2), the virus that causes Corona Virus Disease of 2019 (COVID-19), typically recover to their baseline health status within a few weeks after the acute infection. However, a substantial portion of individuals experience persistent post-infective symptoms (1–3). Persisting symptoms have been reported in patients regardless of the severity of the acute COVID-19 infection (4), and even children and young adults who experienced predominantly mild cases of acute COVID-19 may endure prolonged symptoms (5). These enduring health issues commonly include various neurological complaints such as fatigue, post-exertional malaise, headache, memory difficulties, and sleep disturbances (6–8). The World Health organization (WHO) has defined these long-lasting symptoms following confirmed or suspected COVID-19 infection, with no alternative diagnosis to explain them, as Post-COVID-19 condition (PCC) (9). PCC exhibits significant clinical overlap with post-infective fatigue syndrome (PIFS) (10), and numerous queries concerning the underlying mechanisms of disease and its natural progression still lack definite answers. Further, it is still to be established whether the subjective experience of neurological and neuropsychological symptoms in PCC correspond with objectively measurable deficits (8, 11–13).

Multiple mechanisms have been suggested as potential underlying mechanisms of neurobiological aberrations in COVID-19 and PCC. In the acute and subacute stages, CNS involvement may be due to immune activation triggered by systemic inflammation, microvascular damage, thromboembolic events, or non-specific hypoxic effects resulting from severe illness (14, 15). One mechanism proposed to account for the manifestations of PCC revolves around activation of the neuroimmune system (16, 17). Alternatively, the symptoms of PCC may be explained from functional CNS alterations (18), analogous to common mechanisms of chronic pain conditions (19). This latter explanation acknowledges that symptoms may arise independently of neuronal damage and/or interoceptive afferent signals, and resonates with previous published evidence of psychosocial factors as important predictors of persistent symptoms (20, 21).

The intra-axonal protein neurofilament light chain (Nfl) is a validated biomarker for neuroaxonal injury and neuroinflammation regardless of cause (22–24). Numerous studies have demonstrated a strong correlation between levels of Nfl in cerebrospinal fluid (CSF) and blood serum samples (25, 26) rendering it widely applicable as a biomarker for neuroinflammation and neurodegeneration. Glial fibrillary acidic protein (GFAp) is an astrocytic cytoskeletal protein upregulated in activated astrocytes, recognized for its swift elevation in both CSF and serum in response to acute brain injuries. Studies have evidenced a robust correlation between the levels of GFAp detected in CSF and blood serum samples (27–30). Elevated serum levels of these biomarkers in the acute phase of COVID-19 infection provides evidence of astrocytic and neuroaxonal damage in patients undergoing a severe course of SARS-CoV-2 infection (13, 31–34). We have previously reported these biomarkers to be slightly elevated in the subacute phase of SARS-CoV-2 infection in adolescents and young adults with mildly symptomatic disease (11). Studies examining neuroinflammatory biomarkers during follow-up after COVID-19 infection yield varied outcomes, even when they are limited to mild initial cases (35, 36).

In the current study we report serum levels of NfL and GFAp at 6-This study aimed to investigate brain injury biomarkers, neurocognitive test performance, and self-reported neurological and neuropsychological symptoms in young people with PCC. We examined cross-sectional data from 6-month follow-up of a large prospective cohort of adolescents and young adults with and without COVID-19.

## Methods

### Study design

The long-term effects of COVID-19 in Adolescents (LoTECA) project is a longitudinal observational cohort study of non-hospitalized adolescents and young adults. Participants testing positive and negative for SARS-CoV-2 were included, with follow-up at 6 and 12 months (Clinical Trials ID: NCT04686734). Details of the study design have been described previously (20). This study reports results from the 6-month follow-up visit.

Ethical approval for this project was granted by The Regional Committee for Ethics in Medical Research. Written informed consent was obtained from each participant at study inclusion.

### Participants

Between 24 December 2020 and 18 May 2021, a consecutive cohort of adolescents and young adults undergoing SARS-CoV-2 testing with reverse transcription-polymerase chain reaction (RT-PCR) were enrolled. All participants were recruited from one of two microbiological laboratories, Fürst Medical Laboratory or Department of Microbiology and Infection Control at Akershus University Hospital, both located in Southeast Norway. The prevailing strain of SARS-CoV-2 in this geographical area during most of the recruitment period was B.1.1.7 (Alpha). Any SARS-CoV-2 positive individuals were considered eligible for enrolment after fulfilling a 10-day quarantine. Concurrently, a SARS-CoV-2 negative control group was recruited among individuals exhibiting a similar distribution of sex and age as the SARS-CoV-2 infected cases. Within the SARS-CoV-2 negative group, some individuals had undergone testing due to acute infectious symptoms, while others were asymptomatic close contacts of confirmed cases.

Exclusion criteria at baseline encompassed the following: (1) A duration of more than 28 days since onset of symptoms; (2) Hospitalization due to COVID-19; (3) Pregnancy; and (4) Serological evidence of SARS-CoV-2 infection (in the SARS-CoV-2-negative group).

### Investigational program

At enrolment and each follow-up assessment, all participants attended a comprehensive assessment program at the study center at Akershus University Hospital, Norway. This program encompassed clinical interview, physical examination, blood sample collection, vital sign recording, functional and neurocognitive testing, and completion of questionnaires. The complete investigational program of the LoTECA project has been published previously (20).

### Laboratory assays

Blood samples were collected via antecubital venepuncture as the first part of the 6-month follow-up visit. Samples were subjected to analysis for routine clinical markers. To identify previous infection with COVID-19, serum samples were analyzed for SARS-CoV-2 nucleocapsid and receptor binding antibodies.

Serum samples for measurement of GFAp and Nfl was collected in 3.5 mL Vacuette R (Greiner bio-one GmbH) with gel. Samples underwent clotting for a minimum of 30 min. Within 2 h, they were processed by centrifugation at 2,200 g for 10 min. The aliquots were stored immediately at −80°C until analysis. Serum GFAp and Nfl measurement was conducted at the Clinical Neurochemistry Laboratory, Sahlgrenska University Hospital, Sweden, by certified laboratory technicians blinded to clinical data. The analysis was performed utilizing commercially available Single Molecule Array (Simoa) assays on an HD-X analyzer (Human Neuro 2-plex B assay), as instructed by the manufacturer (Quanterix, Billerica, MA). Calibrators were run in duplicates, while the samples were diluted four-fold and run as singlicates. To monitor assay performance, two quality control (QC) samples, with different concentration levels, were run in duplicates at the beginning and end of each analytical run. For the QC sample with a Nfl concentration of 14.1 pg./mL, repeatability and intermediate precision were both 6.2%, and for QC samples with a concentration of 77.3 pg./mL, both repeatability and intermediate precision was 5.9%. For GFAp CQ samples with concentration 99.4 pg./mL, repeatability was 4.4% and intermediate precision was 8.3%. For GFAp QC samples with concentration 281 pg./mL, repeatability was 5.6% and intermediate precision was 6.3%.

### Neurocognitive testing

During the 6-month follow-up visit, all study participants underwent neurocognitive assessment with two standardized tests: the Digit-Span Test from the Wechsler Intelligence Scale for Children, 4th edition (WISC) (37), and the Hopkins Verbal Learning Test-Revised (HVLT-R) (38).

The Digit-Span Test is a tool for evaluating verbal and auditory working memory. An examiner presents a series of random digits verbally. The initial digit sequence comprises two random numbers, and with each subsequent sequence, an additional digit is included. During the digit span forward mode, the participant is tasked with repeating the digits in the same order as they were presented, while in the digit span backward mode, the digits are to be repeated in reverse order. A score of one point is assigned for each correctly recalled digit sequence. The test is discontinued when the participant provides incorrect responses for two sequences of equal length. Results are reported in the form of sum scores for digit span forward and backward, as well as a total sum score.

The HVLT-R test is designed to assess verbal learning, delayed recall, and recognition. A standardized procedure is followed, where the examiner orally presents a list of 12 words, and the participant is tasked with repeating as many of these words as possible in three consecutive trials. The cumulative score for verbal learning memory is determined by summing the total number of words remembered across the three trials, with a possible range of scores ranging from 0 to 36. To evaluate delayed verbal memory, the number of words successfully recalled after a 20-min interval is recorded; score ranges from 0 to 12. Subsequently, a list of 24 words is presented, of which 12 words are identical to those from the initial list. The number of correctly recognized words and falsely recognized words are recorded separately; scores range from 0 to 12.

### Questionnaires

A questionnaire was employed to gather information regarding comorbidities, family medical history, current medication, smoking habits, substance abuse, physical activity and parental occupation. Parental occupation was used to gauge socioeconomic status.

Sleep problems and pain were recorded through the Karolinska Sleep Inventory and the Brief Pain Inventory, respectively (39, 40). In the Karolinska Sleep Inventory, a total of 12 items addressed frequency of sleep disturbances on 6-point Likert scales, where 1 is “never” and 6 is “all the time”; then, the scoring was reversed, and total sum score was computed across all items ranging from 12 to 72, where *lower* scores indicate more sleep disturbances. Accordingly, indexes for insomnia, awakening problems, and sleepiness were computed as sum scores across relevant items. In the Brief Pain Inventory, a total of four items addressed different aspects of pain on 10-point Likert scales, where 1 is “no pain” and 10 is “worst pain imaginable”; total sum score was computed across all items ranging from 4 to 40, where higher scores indicate more pain.

In addition, five neurological/neurocognitive symptoms were assessed: Concentration difficulty, difficulty making decisions, memory difficulty, feeling confused or disoriented, and headache. The frequency of these symptoms were assessed using five-point Likert scales, ranging from 1 to 5, with options spanning from “never” to “each day/always.”

### SARS-CoV-2 immunization

Data pertaining to vaccination status was acquired through linkage with the Norwegian Immunization Register (41).

### Case definitions

The WHO definition of Post COVID-19 Condition (PCC) (9) and the modified Fukuda-case definition of Post-Infective Fatigue Syndrome (PIFS) (42) were applied and operationalized at 6-month follow-up, as thoroughly described previously (20). In brief, all participants were categorized as either case or non-case in accordance with both definitions. To enhance accuracy, a distinction was drawn between definite and uncertain classifications, considering concurrent medical and psychiatric comorbidities that could potentially account for the reported symptoms. Both clinical findings, laboratory reports and questionnaire data from baseline and at 6-month follow-up were considered in the identification of PCC and PIFS cases. Two medical doctors blinded to the participants’ initial SARS-CoV-2 status conducted the assessment independently.

Participants were stratified into four groups based on COVID status and adherence to PCC criteria as follows: (1) COVID-19 positive individuals who adhered to PCC criteria (COVID+PCC+); (2) COVID-19 positive individuals who did not adhere to PCC criteria (COVID+PCC-); (3) COVID-19 negative individuals who adhered to PCC criteria (COVID-PCC+); and (4) COVID-19 negative individuals who did not adhere to PCC criteria (COVID-PCC-). A similar categorization was undertaken based on adherence to PIFS criteria (PIFS+ or PIFS-).

### Statistical analysis

For cross-sectional comparisons between COVID-19 positive and COVID-19 negative cases, chi-square test and Wilcoxon rank-sum test were applied as appropriate based on distribution of the data. For comparison across the four groups according to COVID status and PCC/PIFS adherence, one-way ANOVA or Kruskal-Wallis tests were used as appropriate. Post-hoc analyses were conducted to investigate differences between groups that exhibited statistically significant results.

Associations between symptoms, neurocognitive test results, neurological findings and the two brain injury biomarkers Nfl and GFAp were investigated using the non-parametrical Spearman’s rho test.

Statistical analyses were executed using Stata Statistical Software: Release 16 (Statacorp LLC, College Station, TX). A significance threshold of *p* < 0.05 was adopted (two-sided test). Bonferroni correction was incorporated in the spearman’s rho test to account for test multiplicity.

## Results

At baseline 509 (404 SARS-CoV-2 positive, 105 SARS-CoV-2 negative) children and young adults were included in the study. A total of 26 participants were lost to follow-up (22 COVID-19 cases and 4 COVID-19 negative controls). Of the COVID-19 negative controls, 16 were excluded from analyses at 6 months due to SARS-CoV-2 infection during the follow-up period, either self-reported or diagnosed from the appearance of plasma SARS-CoV-2 nucleocapsid antibodies. In addition, one COVID-19 case suffering from multiple sclerosis was excluded from the current analysis of neurological involvement. A total of 466 participants (381 SARS-CoV-2 positive, 85 SARS-CoV-2 negative) were included in the final analysis of the present paper.

The median time from baseline visit to follow-up was 193 days for both the SARS-CoV-2 positive and SARS-CoV-2 negative group. An overview of demographics and background characteristics are reported in Table 1.

**Table 1:** Cohort characteristics at 6-month follow-up by SARS-CoV-2 status on inclusion.

Characteristic	Participants, No. (%)		
	SARS-CoV-2 Positive group	SARS-CoV-2 Negative group	*p*-value^1^
	*N* = 381	*N* = 85	
**Background**			
Sex			
Female-N (%)	229 (60)	31 (64)	
Male	152 (40)	54 (36)	0.559^2^
Age at baseline, median (iqr)	17.5 (14.8–21.3)	17.7 (15.3–20.0)	0.655^3^
Days since baseline visit, median (iqr)	193 (188–199)	193 (188–205)	0.473^3^
Immunization against COVID-19	278 (73%)	78 (92%)	<0.001^2^
BMI kg/m2, mean (SD)	23.2 (4.70)	23.2 (4.3)	0.489^2^
Ethnicity			
Caucasian, No. (%)	286 (75%)	83 (98%)	
Other, No. (%)	95 (25%)	2 (2.4%)	<0.001^2^
Chronic disease, self^4^, No. (%)	65 (17%)	17 (20%)	0.541^2^
Chronic disease, family member^4^, No. (%)	122 (33%)	30 (36%)	0.677^2^
ISEI-08 Index of socioeconomy—median (iqr)	60.3 (36.4–75.5)	62.4 (47.3–73.4)	0.517^3^
**Biomarkers**			
B-Hemoglobin g/dL, mean (SD)	13.6 (1.18)	13.6 (1.03)	0.437
B-Leukocytes*10^9^/L, mean (SD)	6.1 (1.76)	5.9 (1.51)	0.878
B-Platelets*10^9^/L, mean (SD)	270 (59)	276 (58)	0.180
**S-CRP** ^**5**^ **mg/L, no (%)**			
<5	354 (95%)	76 (92%)	
>5	19 (5%)	7 (8%)	0.235
P-Ferritin μg/L, median (iqr)	45 (30–76.5)	44 (33–63)	0.809
S-Sodium mmol/L, mean (SD)	139 (1.76)	139 (1.82)	0.676
S-Potassium mmol/L, mean (SD)	4.0 (0.24)	4.1 (0.28)	0.051
P-Creatinine, mean (SD)	67 (13.3)	68 (11.7)	0.384
P-LD U/L, mean (SD)	161 (31.6)	158 (34.1)	0.759
P-ALAT, median (iqr)	17 (13–23)	16 (13–20)	0.146^3^
S-Neurofilament light chain, pg./mL, median (iqr)	4.7(2.1)	4.6(1.8)	0.374^3^
S-Glial fibrillary acidic protein, pg./mL, median (iqr)	65.1(34.8)	70.1(33.8)	0.381^3^
**Caseness**			
PCC cases-no. of cases (%)	184 (48%)	40 (47%)	0.837^2^
PIFS cases-no. of cases (%)	53 (14%)	7 (8%)	0.158^2^

^1^T-test unless otherwise stated. ^2^Chi2 test. ^3^Wilcoxon-rank test. ^4^Self-reported, from questionnaires. ^5^Serum CRP levels are low in the majority of cases. The participants are therefore reported as frequencies within categories, maximum observation of 118 mg/L in COVID-19 positive group.

As previously reported (20), there was no difference in adherence to the WHO post-COVID-19 condition nor in adherence to the criteria of post infectious fatigue syndrome based on previous COVID-19 exposure.

### Comparison according to COVID-19 status and PCC adherence

Results from comparison across the four groups according to COVID status and PCC adherence are presented in Table 2. Significant differences between groups were found for all symptoms of neurocognitive dysfunction, as well as pain and sleep difficulties. *Post-hoc* test results are reported in Supplementary Table 1. Generally, the *post-hoc* tests of reported symptoms showed significant differences between groups that differed in PCC adherence, but not between groups with differences in COVID status and similar PCC adherence.

**Table 2:** Cross-sectional comparison of symptoms, clinical and laboratory findings, and neurocognitive test results among COVID-19 cases and non-COVID controls for participants with and without post-COVID-19 Condition (PCC) at 6 months follow-up.

Reported symptoms	COVID-19 cases		Non-COVID controls		
	COVID + PCC+	COVID + PCC-	COVID-PCC+	COVID-PCC-	*p*-value^a^
**Symptoms suggesting neurocognitive aberrations**					
Concentration difficulty, score-mean (SD)	3.3 (1.2)	1.9 (1.1)	3.3(1.1)	2.1 (1.0)	<0.001
*Confidence interval*	*3.2–3.5*	*1.7–2.0*	*2.9–3.6*	*1.8–2.4*	
Difficulty making decisions, score-mean (SD)	2.7 (1.3)	1.6 (0.9)	2.3 (1.1)	1.6 (0.9)	<0.001
*Confidence interval*	*2.5–2.8*	*1.4–1.7*	*2.0–2.7*	*1.3–1.9*	
Memory difficulty, score-mean (SD)	2.9 (1.3)	1.7 (1.0)	2.6 (1.3)	1.8 (1.2)	<0.001
*Confidence interval*	*2.7–3.1*	*1.6–1.8*	*1.2–3.0*	*1.4–2.1*	
Feeling confused or disoriented, score-mean (SD)	1.8 (1.1)	1.2 (0.6)	1.5 (0.7)	1.2 (0.7)	<0.001
*Confidence interval*	*1.7–2.0*	*1.1–1.3*	*1.3–1.8*	*1.0–1.5*	
**Sleep**					
Karolinska sleep questionnaire, total score-mean (SD)	40.7 (11.1)	55.0 (10.4)	42.8 (8.2)	51.4 (10.0)	<0.001
*Confidence interval*	*39.1–42.3*	*53.5–56.5*	*40.1–45.4*	*48.4–54.4*	
Insomnia subscore, −mean (SD)	14.6 (4.5)	18.7 (3.9)	15.1 (4.0)	17.7 (4.0)	<0.001
*Confidence interval*	*14.0–15.3*	*18.2–19.3*	*13.8–16.4*	*16.5–18.9*	
Awakening problems subscore-mean (SD)	8.9 (3.7)	13.3 (3.6)	9.3 (3.0)	12.2 (3.2)	<0.001
*Confidence interval*	*8.3–9.4*	*12.8–13.8*	*8.3–10.3*	*11.3–13.1*	
Sleepiness subscore, −mean (SD)	14.2 (3.8)	18.8 (3.6)	15.1 (3.0)	17.6 (3.6)	<0.001
*Confidence interval*	*13.6–14.8*	*18.2–19.3*	*14.2–16.1*	*16.5–18.7*	
**Pain**					
Headache, score-mean (SD)	2.6 (1.0)	1.6 (0.8)	2.3 (1.0)	2.0 (0.9)	<0.001
*Confidence interval*	*2.4–2.7*	*1.5–1.8*	*2.0–2.6*	*1.7–2.3*	
Brief pain inventory total score, mean (SD)	11.5 (5.5)	7.7(3.8)	11.7(4.5)	9.7(4.5)	<0.001
*Confidence interval*	*10.8–12.4*	*7.2–8.3*	*10.3–13.1*	*8.4–11.1*	
Worst pain in 24 h, mean (SD)	4.4 (2.3)	3.0 (2.1)	5.2 (2.3)	4.0 (2.3)	<0.001
*Confidence interval*	*4.1–4.8*	*2.7–3.2*	*4.5–5.9*	*3.4–4.7*	
Least pain in 24 h, mean (SD)	1.9 (1.5)	1.4 (1.1)	1.4 (0.7)	1.8 (1.8)	<0.001
*Confidence interval*	*1.7–2.1*	*1.3–1.6*	*1.2–1.6*	*1.2–2.3*	
Pain on average, mean (SD)	3.2 (1.8)	1.9 (1.2)	3.0 (1.4)	2.5 (2.0)	<0.001
*Confidence interval*	*2.9–3.4*	*1.8–2.1*	*2.6–3.5*	*1.9–3.1*	
Pain right now, mean (SD)	2.1 (1.6)	1.4 (1.2)	2.1 (1.3)	1.4 (0.6)	<0.001
*Confidence interval*	*1.9–2.3*	*1.2–1.6*	*1.7–2.5*	*1.2–1.5*	
**Neurological findings and brain injury biomarkers**					
Neurological examination, any findings -No. (%)	5 (2.7%)	8 (4%)	2(5%)	3(6.7%)	0.625
Neurofilament light chain, pg./mL, median (iqr)	4.5 (1.9)	4.9 (2.5)	4.4 (1.9)	4.6 (1.9)	0.156^b^
*Confidence interval*	*4.2–4.8*	*4.6–5.1*	*4.1–5.0*	*4.4–5.1*	
Glial fibrillary acidic protein, pg./mL, median (iqr)	64.9 (30.3)	65.8 (40.4)	62.2 (30.4)	71.8 (35.9)	0.607^b^
*Confidence interval*	*60.2–68.8*	*61.1–72.8*	*57.9–77.7*	*59.8–86.7*	
**Neurocognitive test results**					
Digit span forward, total sum score -mean (SD)	10.0 (2.5)	9.9 (2.5)	9.7 (2.2)	9.3 (2.4)	0.344
*Confidence interval*	*9.6–10.4*	*9.6–10.3*	*9.0–10.4*	*8.6–10.0*	
Digit span backward, total sum score -mean (SD)	6.0 (2.4)	6.1 (2.2)	6.0 (3.1)	5.6 (1.8)	0.670
*Confidence interval*	*5.7–6.4*	*5.7–6.4*	*5.0–6.9*	*5.0–6.1*	
Digit span summary score, −mean (SD)	16.0 (4.3)	16.0 (4.0)	16.0 (4.0)	14.8 (3.8)	0.368
*Confidence interval*	*15.4–16.6*	*15.4–16.5*	*14.2–17.2*	*13.7–16.0*	
HVLT-R immediate recall, total sum score -mean (SD)	23.4 (4.8)	23.9 (4.4)	22.9 (5.2)	23.0 (4.6)	0.401
*Confidence interval*	*22.7–24.1*	*23.3–24.5*	*21.2–24.6*	*21.6–24.3*	
HVLT-R delayed recall, total sum score -mean (SD)	8.3 (2.3)	8.3 (2.2)	7.9 (2.5)	7.9 (2.5)	0.521
*Confidence interval*	*8.0–8.7*	*8.0–8.6*	*7.1–8.7*	*7.2–8.7*	
HVLT-R correct recognition, mean (SD)	11.4 (1.1)	11.4 (0.9)	11.4 (0.8)	11.4 (1.0)	0.941
*Confidence interval*	*11.3–11.6*	*11.3–11.6*	*11.3–11.6*	*11.1–11.7*	
HVLT-R false recognition, mean (SD)	0.3 (0.7)	0.3 (0.6)	0.4 (0.7)	0.5 (0.9)	0.349
*Confidence interval*	*0.2–0.4*	*0.2–0.4*	*0.1–0.6*	*0.2–0.8*	

^a^One-way ANOVA unless otherwise stated. ^b^Kruskal-Wallis.

**Figure 1:**
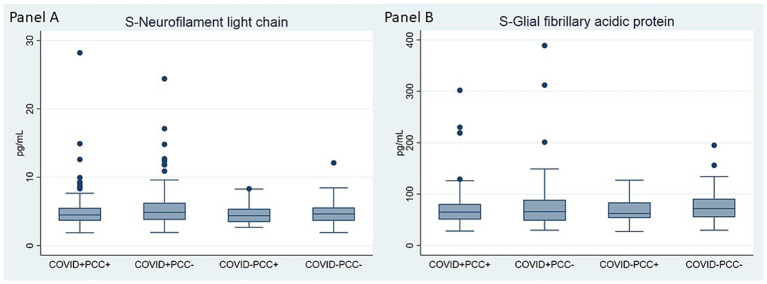
Comparison of serum levels of neurofilament light chain **(A)** and glial fibrillary acidic protein **(B)** at 6-months follow-up within groups of COVID-19 status and PCC adherence. Kruskal-Wallis test was conducted to examine the differences between groups. Panel **(A)**: Chi square = 5.22, *p* = 0.156, df = 3; Panel **(B)**: Chi square = 1.84, *p* = 0.607, df = 3.

**Figure 2:**
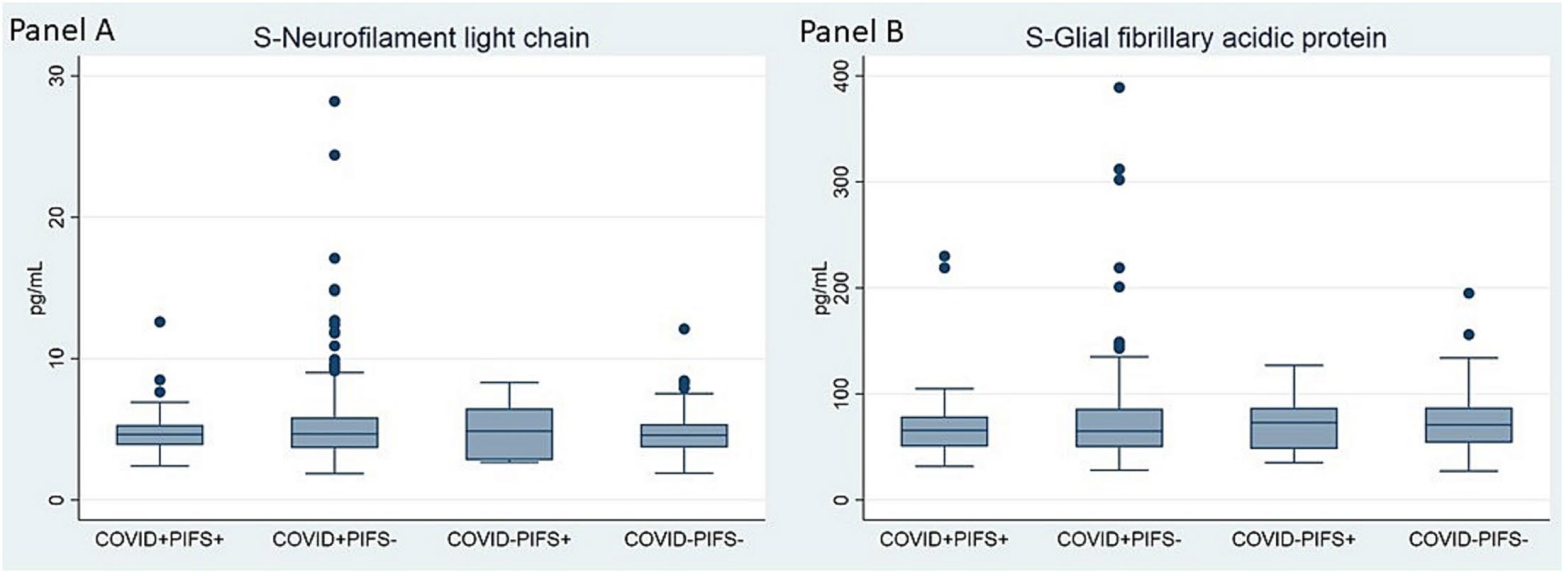
Comparison of serum levels of neurofilament light chain **(A)** and glial fibrillary acidic protein **(B)** at 6-months follow-up within groups of COVID-19 status and PIFS adherence. Kruskal-Wallis test was conducted to examine the differences between groups Panel **(A)**: Chi square = 0.89, *p* = 0.827, df = 3; Panel **(B)**: Chi square = 0.87, *p* = 0.834, df = 3.

Brain injury biomarkers, neurocognitive test performance, and clinical neurological findings did not differ across the four groups (Table 2; Figure 1). Similarly, there were no differences across four groups stratified according to COVID-19 status and adherence to PIFS criteria (Figure 2; Supplementary Tables 2, 3).

### Associations to PCC within the SARS-CoV-2 positive cohort

**Figure 3:**
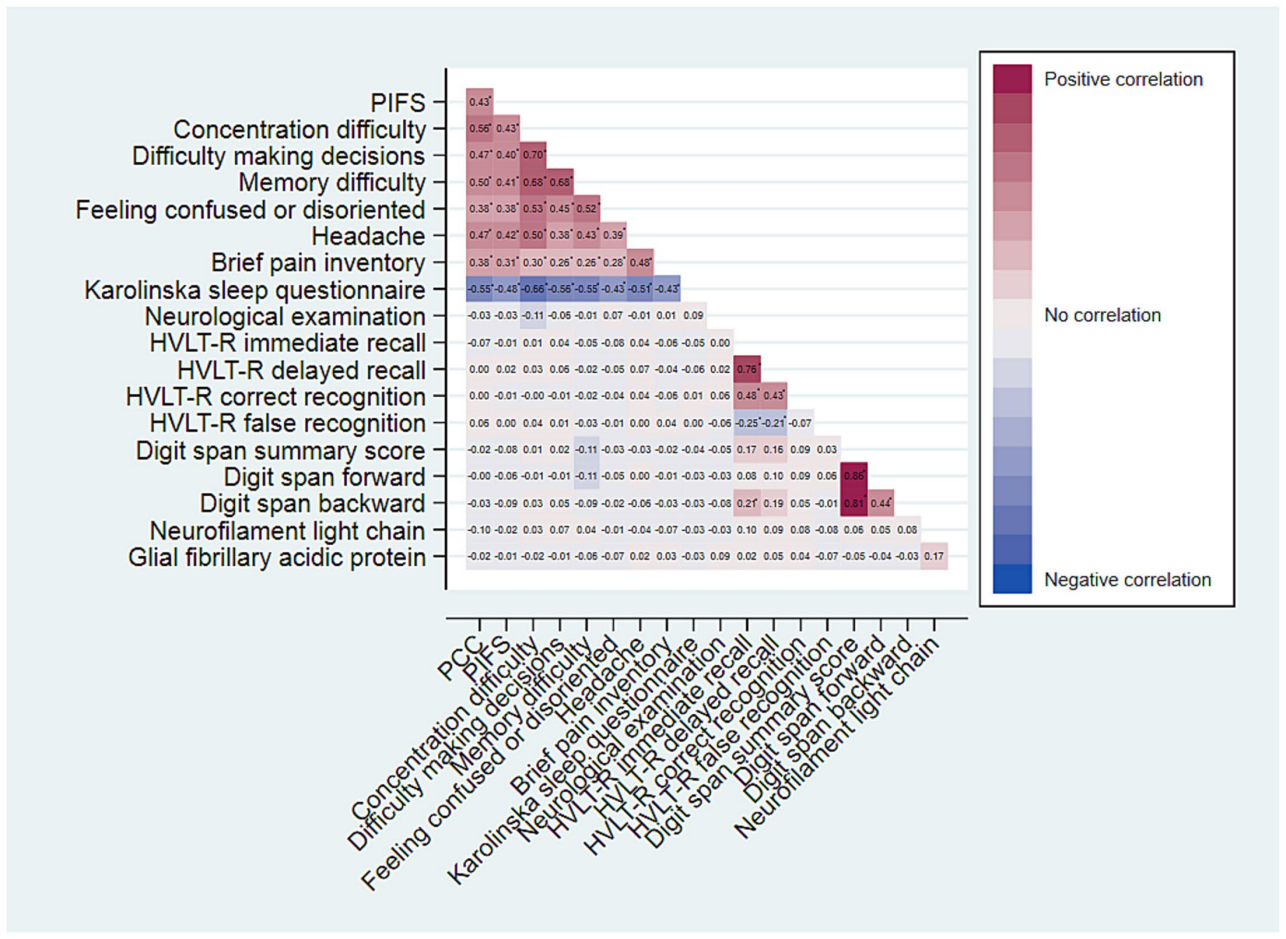
Heatplot of Spearman’s Rho correlation coefficients for variables of neurocognitive symptoms, neurocognitive test results and adherence to PCC and PIFS, respectively. Coefficients marked with *, are significant at a Bonferroni adjusted significance level of α = 0.05/162 = 0.0003.

Associations between PCC, PIFS, as well as subjectively reported symptoms, brain injury biomarkers, and neurocognitive test results are presented in Figure 3. Neither of the biomarkers, Nfl or GFAp, demonstrated any association with PCC nor PIFS, nor were they associated with the reported symptoms or neurocognitive test results. In contrast, both PCC and PIFS exhibited significant association with any subjective symptoms of pain, sleep disturbances, memory issues, difficulty concentrating, decision-making challenges and feeling confused or disorientated. However, none of the subjective symptoms was associated with neurocognitive test results. Complete overview of correlation coefficients and significance levels from the spearman’s rho test are provided in Supplementary Table 4.

## Discussion

In the present study of a large group of young, non-hospitalized COVID-19 convalescents, the main findings were: (a) That brain injury biomarkers were normalized 6 months after acute infection; (b) That neither brain injury biomarkers, neurocognitive test performance nor clinical neurological finding were associated with PCC or PIFS; and (c) That the burden of subjective neurological and/or neuropsychological symptoms is high in both PCC and PIFS.

In baseline data from our cohort, we observed a slight increase in Nfl and GFAp levels in the sub-acute phase of COVID-19 (11). Our current finding of these brain injury biomarkers returning to normal levels 6-months after mild COVID-19 infection aligns with the findings of others. Kanberg et al. found that Nfl and GFAp serum concentrations were normalized 6 months post-infection in a cohort of mild, moderate and severe COVID-19 cases (43), and Rogatzki et al. found normalization of serum levels of Nfl/GFAp as early as 1 month following mild COVID-19 infection in young adults (35).

Both Nfl and GFAp has previously been suggested as useful biomarkers for identification of patient suffering from neurological sequelae following COVID-19 infection (44). In a study of critically ill COVID-19-patients investigated 3 to 6 months after discharge from the intensive care unit, GFAp and Nfl were found to be associated with neurocognitive dysfunction and neuropsychiatric outcome (45). Contrary, in our cohort of young individuals with mild disease course, there were no association between GFAp/Nfl and neurocognitive symptoms or post-COVID-19 symptomatology. This corroborates with previous reports on milder cases. De Boni et al. reported lower levels of Nfl and GFAp in patients with persistent post-COVID-19 headache compared to patients with severe COVID-19 (46). Lennol et al. evidenced normalization of plasma GFAp and Nfl within 2 months following acute infection in patients with or without symptoms of fatigue, headache and memory loss (47), and Farhadian et al. found no evidence of neuroinflammation or blood–brain barrier dysfunction in a cohort of adults with self-reported PCC (48).

Our results are in contrast to those of Telser et al. (36) who reported higher GFAp levels among participants adhering to PCC criteria compared to those without PCC adherence. However, the study by Telser et al. is limited by the rather small sample size of 146 COVID-19 positive participants and the lack of a COVID-19 negative control group. Additionally, the patients’ COVID-19 status was determined retrospectively based on the presence of antibodies, and differences in time span to the acute infection were not accounted for.

In line with previous reports, the present data confirm a high burden of neurological and/or neuropsychiatric symptoms among patients with PCC and PIFS (49, 50). However, these previous reports do not include measures of neuronal damage nor objective testing of neurocognitive performance. A striking result from the present study is the discrepancy between subjective symptoms and objective findings. This resonates with findings from PIFS in the aftermath of other infections (51, 52).

Various hypotheses have been suggested to explain the pathogenesis of PCC. These include systemic chronic inflammation (53), as well as neuroinflammation and autoimmunity (54). In severe cases of acute COVID-19 there is evidence that the neuroinflammation is linked to cytokine storms, as elevated serum Nfl and GFAp are associated with elevations in pro-inflammatory cytokines (55, 56). Elevated levels of Nfl have even been found to have prognostic value in acute, severe cases of COVID-19 (57). The normalization of brain injury biomarkers 6 months after mild COVID-19 infection found in the current study, suggests that the neurological symptoms associated with PCC do not align with enduring or ongoing CNS injury. This argue against the notion of neuroinflammation as an explanation for the persisting symptoms. In a previous publication (58), we reported a distinct immune signature associated with COVID-19 at 6-month follow-up. However, this did not appear to be connected to PCC symptomatology. In the current study, we found no evidence of ongoing neuroinflammation, and neither brain injury biomarkers nor neurocognitive test results were associated with subjective reported symptomatology. Hence, the findings from the present study adds to a growing body of evidence suggesting that PCC may be associated with functional CNS alterations and have origins more related to a combination of biological, psychological and social factors, rather than being solely biomedical in nature (59).

The small number of COVID-19 negative controls is a limitation to the study. Further, controls were recruited following SARS-CoV-2 testing for either infectious symptoms, or suspected SARS-CoV-2 exposure. Other viral diseases could have caused the symptoms leading to testing. Considering the established role of Epstein–Barr virus (EBV) as a trigger for post-infectious fatigue syndrome (60), individuals with recent EBV infection were not included in the analysis. To further strengthen the quality of the control group, SARS-CoV-2 antibody testing was conducted both at inclusion, and 6 months follow-up. Participant displaying antibodies indicative of prior COVID-19 infection were excluded from the control group.

The external validity of the study is limited by the potential for self-selection bias. It is plausible that our participants exhibited a higher prevalence of symptoms compared to the general population. This selection bias might be even more relevant in the control group. Further, the current study only focused on a cohort of young individuals, mostly infected with the B1.1.7 variant of SARS-CoV-2. Therefore, the generalizability of our findings to other viral strains, and to older age groups, who could exhibit increased vulnerability to both COVID-19 and PCC remains uncertain.

## Conclusion

In the current study, we found that brain injury biomarkers were normalized 6 months after acute COVID-19 and that the post-COVID-19 condition, despite high symptom burden, was not associated with brain injury biomarkers, neurocognitive test performance or clinical neurological symptoms. Hence, among adolescents and young adults, neurological symptoms linked to post-COVID-19 condition do not align with continuous CNS damage, thereby challenging the notion of neuroinflammation as an underlying cause of the enduring symptoms.

## Data availability statement

The raw data supporting the conclusions of this article will be made available by the authors, without undue reservation.

## Ethics statement

The studies involving humans were approved by the Regional Committee for Ethics in Medical Research. The studies were conducted in accordance with the local legislation and institutional requirements. Written informed consent for participation in this study was provided by the participants’ legal guardians/next of kin.

## Author contributions

LH: Conceptualization, Data curation, Formal analysis, Investigation, Methodology, Writing – original draft. JS: Conceptualization, Data curation, Formal analysis, Investigation, Methodology, Writing – review & editing. LL: Conceptualization, Data curation, Investigation, Methodology, Writing – review & editing. TS-S: Data curation, Investigation, Methodology, Writing – review & editing. HZ: Writing – review & editing. KB: Data curation, Writing – review & editing. TH: Conceptualization, Writing – review & editing, Data curation. VW: Conceptualization, Funding acquisition, Investigation, Methodology, Project administration, Resources, Supervision, Writing – review & editing.

## References

[1] Huang C Huang L Wang Y Li X Ren L Gu X. 6-month consequences of COVID-19 in patients discharged from hospital: a cohort study. Lancet. (2021) 397:220–32. doi: 10.1016/S0140-6736(20)32656-8, PMID: 33428867 PMC7833295

[2] Huang L Yao Q Gu X Wang Q Ren L Wang Y. 1-year outcomes in hospital survivors with COVID-19: a longitudinal cohort study. Lancet. (2021) 398:747–58. doi: 10.1016/S0140-6736(21)01755-4, PMID: 34454673 PMC8389999

[3] Al-Aly Z Xie Y Bowe B. High-dimensional characterization of post-acute sequelae of COVID-19. Nature. (2021) 594:259–64. doi: 10.1038/s41586-021-03553-9, PMID: 33887749

[4] Logue JK Franko NM McCulloch DJ McDonald D Magedson A Wolf CR. Sequelae in adults at 6 months after COVID-19 infection. JAMA Netw Open. (2021) 4:e210830. doi: 10.1001/jamanetworkopen.2021.0830, PMID: 33606031 PMC7896197

[5] Haddad A Janda A Renk H Stich M Frieh P Kaier K. Long COVID symptoms in exposed and infected children, adolescents and their parents one year after SARS-CoV-2 infection: a prospective observational cohort study. EBioMedicine. (2022) 84:104245. doi: 10.1016/j.ebiom.2022.104245, PMID: 36155957 PMC9495281

[6] Premraj L Kannapadi NV Briggs J Seal SM Battaglini D Fanning J. Mid and long-term neurological and neuropsychiatric manifestations of post-COVID-19 syndrome: a meta-analysis. J Neurol Sci. (2022) 434:120162. doi: 10.1016/j.jns.2022.120162, PMID: 35121209 PMC8798975

[7] Molteni E Sudre CH Canas LS Bhopal SS Hughes RC Antonelli M. Illness duration and symptom profile in symptomatic UK school-aged children tested for SARS-CoV-2. Lancet Child Adolesc Health. (2021) 5:708–18. doi: 10.1016/S2352-4642(21)00198-X, PMID: 34358472 PMC8443448

[8] Pinzon RT Wijaya VO Jody AA Nunsio PN Buana RB. Persistent neurological manifestations in long COVID-19 syndrome: a systematic review and meta-analysis. J Infect Public Health. (2022) 15:856–69. doi: 10.1016/j.jiph.2022.06.013, PMID: 35785594 PMC9221935

[9] Soriano JB Murthy S Marshall JC Relan P Diaz JV WHO Clinical Case Definition Working Group on Post-COVID-19 Condition. A clinical case definition of post-COVID-19 condition by a Delphi consensus. Lancet Infect Dis. (2022) 22:e102–7. doi: 10.1016/S1473-3099(21)00703-9, PMID: 34951953 PMC8691845

[10] Wong TL Weitzer DJ. Long COVID and Myalgic encephalomyelitis/chronic fatigue syndrome (ME/CFS)—a systemic review and comparison of clinical presentation and symptomatology. Medicina. (2021) 57:418. doi: 10.3390/medicina57050418, PMID: 33925784 PMC8145228

[11] Havdal LB Berven LL Selvakumar J Stiansen-Sonerud T Leegaard TM Tjade T. Neurological involvement in COVID-19 among non-hospitalized adolescents and young adults. Front Neurol. (2022) 13:915712. doi: 10.3389/fneur.2022.915712, PMID: 35812102 PMC9257204

[12] Virhammar J Nääs A Fällmar D Cunningham JL Klang A Ashton NJ. Biomarkers for central nervous system injury in cerebrospinal fluid are elevated in COVID-19 and associated with neurological symptoms and disease severity. Eur J Neurol. (2021) 28:3324–31. doi: 10.1111/ene.14703, PMID: 33369818 PMC8518988

[13] Kanberg N Ashton NJ Andersson LM Yilmaz A Lindh M Nilsson S. Neurochemical evidence of astrocytic and neuronal injury commonly found in COVID-19. Neurology. (2020) 95:e1754–9. doi: 10.1212/WNL.0000000000010111 32546655

[14] Franke C Ferse C Kreye J Reincke SM Sanchez-Sendin E Rocco A. High frequency of cerebrospinal fluid autoantibodies in COVID-19 patients with neurological symptoms. Brain Behav Immun. (2021) 93:415–9. doi: 10.1016/j.bbi.2020.12.022, PMID: 33359380 PMC7834471

[15] Solomon T. Neurological infection with SARS-CoV-2 — the story so far. Nat Rev Neurol. (2021) 17:65–6. doi: 10.1038/s41582-020-00453-w, PMID: 33414554 PMC7789883

[16] Hanson BA Visvabharathy L Ali ST Kang AK Patel TR Clark JR. Plasma biomarkers of Neuropathogenesis in hospitalized patients with COVID-19 and those with Postacute sequelae of SARS-CoV-2 infection. Neurol Neuroimmunol Neuroinflamm. (2022) 9:e1151. doi: 10.1212/NXI.0000000000001151, PMID: 35256481 PMC8901169

[17] Pröbstel AK Schirmer L. SARS-CoV-2-specific neuropathology: fact or fiction? Trends Neurosci. (2021) 44:933–5. doi: 10.1016/j.tins.2021.10.006, PMID: 34716032 PMC8519811

[18] Van den Bergh O Witthöft M Petersen S Brown RJ. Symptoms and the body: taking the inferential leap. Neurosci Biobehav Rev. (2017) 74:185–203. doi: 10.1016/j.neubiorev.2017.01.015, PMID: 28108416

[19] Barrett LF Simmons WK. Interoceptive predictions in the brain. Nat Rev Neurosci. (2015) 16:419–29. doi: 10.1038/nrn3950, PMID: 26016744 PMC4731102

[20] Selvakumar J Havdal LB Drevvatne M Brodwall EM Lund Berven L Stiansen-Sonerud T. Prevalence and characteristics associated with post-COVID-19 condition among nonhospitalized adolescents and young adults. JAMA Netw Open. (2023) 6:e235763. doi: 10.1001/jamanetworkopen.2023.5763, PMID: 36995712 PMC10064252

[21] Wang S Quan L Chavarro JE Slopen N Kubzansky LD Koenen KC. Associations of depression, anxiety, worry, perceived stress, and loneliness prior to infection with risk of post–COVID-19 conditions. JAMA Psychiatry. (2022) 79:1081–91. doi: 10.1001/jamapsychiatry.2022.2640, PMID: 36069885 PMC9453634

[22] Fyfe I. Neurofilament light chain — new potential for prediction and prognosis. Nat Rev Neurol. (2019) 15:557–7. doi: 10.1038/s41582-019-0265-2, PMID: 31506590

[23] Zetterberg H. Is there a value of Neurofilament light as a biomarker for neurodegeneration in Parkinson's disease? Mov Disord. (2020) 35:1111–2. doi: 10.1002/mds.28101, PMID: 32691913

[24] Abu-Rumeileh S Abdelhak A Foschi M D'Anna L Russo M Steinacker P. The multifaceted role of neurofilament light chain protein in non-primary neurological diseases. Brain. (2023) 146:421–37. doi: 10.1093/brain/awac328, PMID: 36083979 PMC9494370

[25] Hendricks R Baker D Brumm J Davancaze T Harp C Herman A. Establishment of neurofilament light chain Simoa assay in cerebrospinal fluid and blood. Bioanalysis. (2019) 11:1405–18. doi: 10.4155/bio-2019-0163, PMID: 31401845

[26] Lee Y Lee BH Yip W Chou P Yip BS. Neurofilament proteins as prognostic biomarkers in neurological disorders. Curr Pharm Des. (2020) 25:4560–9. doi: 10.2174/1381612825666191210154535 31820696

[27] Cabezas JA Bustamante A Giannini N Pecharroman E Katsanos AH Tsivgoulis G. Discriminative value of glial fibrillar acidic protein (GFAP) as a diagnostic tool in acute stroke. Individual patient data meta-analysis. J Investig Med. (2020) 68:1379–85. doi: 10.1136/jim-2020-001432, PMID: 32907910

[28] Laverse E Guo T Zimmerman K Foiani MS Velani B Morrow P. Plasma glial fibrillary acidic protein and neurofilament light chain, but not tau, are biomarkers of sports-related mild traumatic brain injury. Brain Commun. (2020) 2:fcaa137. doi: 10.1093/braincomms/fcaa137 33543129 PMC7846133

[29] Watanabe M Nakamura Y Michalak Z Isobe N Barro C Leppert D. Serum GFAP and neurofilament light as biomarkers of disease activity and disability in NMOSD. Neurology. (2019) 93:e1299–311. doi: 10.1212/WNL.0000000000008160, PMID: 31471502

[30] Abdelhak A Foschi M Abu-Rumeileh S Yue JK D’Anna L Huss A. Blood GFAP as an emerging biomarker in brain and spinal cord disorders. Nat Rev Neurol. (2022) 18:158–72. doi: 10.1038/s41582-021-00616-3, PMID: 35115728

[31] Sutter R Hert L de Marchis GM Twerenbold R Kappos L Naegelin Y. Serum Neurofilament light chain levels in the intensive care unit: comparison between severely ill patients with and without coronavirus disease 2019. Ann Neurol. (2021) 89:610–6. doi: 10.1002/ana.26004, PMID: 33377539

[32] Aamodt AH Høgestøl EA Popperud TH Holter JC Dyrhol-Riise AM Tonby K. Blood neurofilament light concentration at admittance: a potential prognostic marker in COVID-19. J Neurol. (2021) 268:3574–83. doi: 10.1007/s00415-021-10517-6, PMID: 33743046 PMC7980743

[33] Sahin BE Celikbilek A Kocak Y Saltoglu GT Konar NM Hizmali L. Plasma biomarkers of brain injury in COVID-19 patients with neurological symptoms. J Neurol Sci. (2022) 439:120324. doi: 10.1016/j.jns.2022.120324, PMID: 35752131 PMC9212259

[34] Abdelhak A Barba L Romoli M Benkert P Conversi F D’Anna L. Prognostic performance of blood neurofilament light chain protein in hospitalized COVID-19 patients without major central nervous system manifestations: an individual participant data meta-analysis. J Neurol. (2023) 270:3315–28. doi: 10.1007/s00415-023-11768-1, PMID: 37184659 PMC10183689

[35] Rogatzki MJ Szeghy RE Stute NL Province VM Augenreich MA Stickford JL. Plasma UCHL1, GFAP, tau, and NfL are not different in young healthy persons with mild COVID-19 symptoms early in the pandemic: a pilot study. Neurotrauma Rep. (2023) 4:330–41. doi: 10.1089/neur.2023.0014, PMID: 37284701 PMC10240333

[36] Telser J Grossmann K Weideli OC Hillmann D Aeschbacher S Wohlwend N. Concentrations of serum brain injury biomarkers following SARS-CoV-2 infection in individuals with and without long-COVID-results from the prospective population-based COVI-GAPP study. Diagnostics. (2023) 13:2167. doi: 10.3390/diagnostics13132167 37443561 PMC10340196

[37] Grizzle R. Wechsler intelligence scale for children In: Goldstein, S., Naglieri, J.A. (eds) Encyclopedia of Child Behavior and Development. 4th ed Springer, Boston, MA. (2011). 1553–5. 10.1007/978-0-387-79061-9_3066

[38] Benedict RHB Schretlen D Groninger L Brandt J. Hopkins verbal learning test – revised: normative data and analysis of inter-form and test-retest reliability. Clin Neuropsychol. (2010) 12:43–55. doi: 10.1076/clin.12.1.43.1726

[39] Klepstad P Loge JH Borchgrevink PC Mendoza TR Cleeland CS Kaasa S. The Norwegian brief pain inventory questionnaire. J Pain Symptom Manag. (2002) 24:517–25. doi: 10.1016/S0885-3924(02)00526-2 12547051

[40] Åkerstedt T Ingre M Kecklund G Folkard S Axelsson J. Accounting for partial sleep deprivation and cumulative sleepiness in the three-process model of alertness regulation. Chronobiol Int. (2009) 25:309–19. doi: 10.1080/07420520802110613 18484366

[41] Norwegian Institute of Public Health, Norwegian immunisation registry SYSVAK. (n.d.). Available online at: https://www.fhi.no/en/hn/health-registries/norwegian-immunisation-registry-sysvak/ (Accessed ongoing at time of follow-up visits)

[42] Fukuda K Straus SE Hickie I Sharpe MC Dobbins JG Komaroff A. The chronic fatigue syndrome: a comprehensive approach to its definition and study. International chronic fatigue syndrome study group. Ann Intern Med. (1994) 121:953–9. doi: 10.7326/0003-4819-121-12-199412150-00009, PMID: 7978722

[43] Kanberg N Simrén J Edén A Andersson LM Nilsson S Ashton NJ. Neurochemical signs of astrocytic and neuronal injury in acute COVID-19 normalizes during long-term follow-up. EBioMedicine. (2021) 70:103512. doi: 10.1016/j.ebiom.2021.103512, PMID: 34333238 PMC8320425

[44] Comeau D Martin M Robichaud GA Chamard-Witkowski L. Neurological manifestations of post-acute sequelae of COVID-19: which liquid biomarker should we use? Front Neurol. (2023) 14:1233192. doi: 10.3389/fneur.2023.1233192, PMID: 37545721 PMC10400889

[45] Bark L Larsson IM Wallin E Simrén J Zetterberg H Lipcsey M. Central nervous system biomarkers GFAp and NfL associate with post-acute cognitive impairment and fatigue following critical COVID-19. Sci Rep. (2023) 13:13144. doi: 10.1038/s41598-023-39698-y, PMID: 37573366 PMC10423244

[46] de Boni L Odainic A Gancarczyk N Kaluza L Strassburg CP Kersting XAK. No serological evidence for neuronal damage or reactive gliosis in neuro-COVID-19 patients with long-term persistent headache. Neurol Res Pract. (2022) 4:53. doi: 10.1186/s42466-022-00217-5, PMID: 36310154 PMC9618412

[47] Lennol MP Ashton NJ Moreno-Pérez O García-Ayllón MS Ramos-Rincon JM Andrés M. Transient changes in the plasma of astrocytic and neuronal injury biomarkers in COVID-19 patients without neurological syndromes. Int J Mol Sci. (2023) 24:2715. doi: 10.3390/ijms24032715, PMID: 36769057 PMC9917569

[48] Farhadian SF Reisert HD McAlpine L Chiarella J Kosana P Yoon J. Self-reported neuropsychiatric post–COVID-19 condition and CSF markers of Neuroinflammation. JAMA Netw Open. (2023) 6:e2342741–1. doi: 10.1001/jamanetworkopen.2023.42741, PMID: 37948085 PMC10638645

[49] Søraas A Bø R Kalleberg KT Støer NC Ellingjord-Dale M Landrø NI. Self-reported memory problems 8 months after COVID-19 infection. JAMA Netw Open. (2021) 4:e2118717. doi: 10.1001/jamanetworkopen.2021.18717, PMID: 34323987 PMC8322992

[50] Brunvoll SH Nygaard AB Fagerland MW Holland P Ellingjord-Dale M Dahl JA. Post-acute symptoms 3-15 months after COVID-19 among unvaccinated and vaccinated individuals with a breakthrough infection. Int J Infect Dis. (2023) 126:10–3. doi: 10.1016/j.ijid.2022.11.009, PMID: 36375693 PMC9651990

[51] Øie MG Rødø ASB Bølgen MS Pedersen M Asprusten TT Wyller VBB. Subjective and objective cognitive function in adolescent with chronic fatigue following Epstein-Barr virus infection. J Psychosom Res. (2022) 163:111063. doi: 10.1016/j.jpsychores.2022.111063, PMID: 36327530

[52] Sandler C Cvejic E Valencia BM Li H Hickie IB Lloyd AR. Predictors of chronic fatigue syndrome and mood disturbance after acute infection. Front Neurol. (2022) 13:935442. doi: 10.3389/fneur.2022.935442, PMID: 35959390 PMC9359311

[53] Su Y Yuan D Chen DG Ng RH Wang K Choi J. Multiple early factors anticipate post-acute COVID-19 sequelae. Cell. (2022) 185:881–895.e20. doi: 10.1016/j.cell.2022.01.014, PMID: 35216672 PMC8786632

[54] Monje M Iwasaki A. The neurobiology of long COVID. Neuron. (2022) 110:3484–96. doi: 10.1016/j.neuron.2022.10.006, PMID: 36288726 PMC9537254

[55] Needham EJ Ren AL Digby RJ Norton EJ Ebrahimi S Outtrim JG. Brain injury in COVID-19 is associated with dysregulated innate and adaptive immune responses. Brain. (2022) 145:4097–107. doi: 10.1093/brain/awac321, PMID: 36065116 PMC9494359

[56] Tsagkaris C Bilal M Aktar I Aboufandi Y Tas A Aborode AT. Cytokine storm and neuropathological alterations in patients with neurological manifestations of COVID-19. Curr Alzheimer Res. (2022) 19:641–57. doi: 10.2174/1567205019666220908084559, PMID: 36089786

[57] De Lorenzo R Loré NI Finardi A Mandelli A Cirillo DM Tresoldi C. Blood neurofilament light chain and total tau levels at admission predict death in COVID-19 patients. J Neurol. (2021) 268:4436–42. doi: 10.1007/s00415-021-10595-6, PMID: 33973106 PMC8108733

[58] Sommen SL Havdal LB Selvakumar J Einvik G Leegaard TM. Inflammatory markers and pulmonary function in adolescents and young adults 6 months after mild COVID-19. Front Immunol. (2022) 13:1081718. doi: 10.3389/fimmu.2022.1081718 36685555 PMC9853911

[59] Saunders C Sperling S Bendstrup E. A new paradigm is needed to explain long COVID. Lancet Respir Med. (2023) 11:e12–3. doi: 10.1016/S2213-2600(22)00501-X, PMID: 36620963

[60] Hickie I Davenport T Wakefield D Vollmer-Conna U Cameron B Vernon SD. Post-infective and chronic fatigue syndromes precipitated by viral and non-viral pathogens: prospective cohort study. BMJ. (2006) 333:575. doi: 10.1136/bmj.38933.585764.AE, PMID: 16950834 PMC1569956

